# Calcium signaling-mediated phosphorylation controls zinc allocation in Arabidopsis

**DOI:** 10.1007/s44154-025-00276-z

**Published:** 2025-12-31

**Authors:** Yanjun Fang, Fangru Liu, Chenyue Yang, Xuening Ma, Cun Wang, Zhenqian Zhang, Chuanfeng Ju

**Affiliations:** 1 State Key Laboratory for Crop Stress Resistance and High-Efficiency Production, College of Life Sciences, Northwest A & F University, Yangling, Shaanxi 712100 China; 2 Institute of Future Agriculture, Northwest A & F University, Yangling, Shaanxi 712100 China

**Keywords:** Calcium signaling, Phosphorylation, CBL-CIPK, ZIP2, Arabidopsis

## Abstract

Zinc (Zn) deficiency in soil can directly result in Zn deficiency in crops, subsequently causing Zn deficiency in humans. Currently, the physiological adaptation mechanisms by which plants respond to Zn deficiency have been fairly well characterized. However, the regulatory mechanisms governing Zn transport in plants remain poorly understood. In this study, we found that CBL1/4/5/8/9-CIPK3/9/23/26 complexes interact with the Zn transporter ZIP2 and phosphorylate its Ser190 residue. Biochemical analyses and complementation experiments in yeast and plants demonstrated that the Ser190 site is essential for the transport activity of ZIP2, and that the Zn transporter ZIP2 is involved in the transport of Zn between the columnar sheath cells in the roots. Notably, the hybrid complementation lines carrying CBL-CIPK-mediated phosphorylation sites of ZIP2 and ZIP12 exhibited enhanced tolerance to Zn deficiency. Overall, these findings suggest that CBL-CIPK-ZIP2/ZIP12 phosphorylation network coordinates Zn allocation in Arabidopsis, providing a potential target for improving Zn deficiency and developing Zn-enriched crop varieties.

**Supplementary Information:**

The online version contains supplementary material available at 10.1007/s44154-025-00276-z.

## Introduction

Zinc (Zn) is an indispensable nutrient for life forms, serving as a structural cofactor for over 300 enzymes and numerous proteins. Approximately 6% of prokaryotic and 9% of eukaryotic proteins are Zn-binding, highlighting its extensive role in biological processes (Sinclair and Krämer 2012; Clemens 2022; Wang et al. 2024). Zn is predominantly stored in the Earth's crust within Zn-rich polymetallic rocks and ores. In calcareous and sandy soils with elevated pH levels, the adsorption capacity of Zn diminishes, resulting in decreased effective Zn content and a propensity for soil Zn deficiency (Zeng et al. 2021; Natasha et al. 2022). This soil Zn deficiency can directly translate to Zn-deficiency crops, ultimately leading to Zn deficiency in humans. The global prevalence of 'hidden hunger' due to Zn deficiency constitutes a significant food nutrition and health issue, affecting over 30% of the world's population and causing more than 400,000 deaths annually (Skalny et al. 2021; Natasha et al. 2022; Stanton et al. 2022; Thiébaut and Hanikenne 2022).

Recently, notable advancements have been made in the study of Zn signaling transduction in plants. Specifically, the F-bZIP transcription factors, *bZIP19* and *bZIP23*, have been characterized as Zn sensors. The Arabidopsis* bzip19bzip23* double mutant exhibits heightened sensitivity to Zn deficiency (Assunção et al. 2010; Inaba et al. 2015). Under Zn-sufficient conditions, bZIP19/23 bind Zn and become inactivated, thereby repressing their transcriptional regulation of Zn-responsive genes. Conversely, Under Zn-deficiency conditions, the dimerized transcription factors bZIP19 and bZIP23 bind to Zn deficiency response elements (ZDREs) in the promoters of their target genes, thereby activating their transcription (Assunção et al. 2010; Lilay et al. 2021). However, compared to macronutrients such as N, P, and K, as well as the micronutrient Fe, the mechanisms underlying Zn perception and signaling transduction in plants remain relatively under-explored.

The ZIP family (ZRT/IRT-like Protein) constitutes the primary system for Zn uptake and transport in plants. However, due to functional redundancy and the scarcity of biochemical evidence, our understanding of ZIP remains fragmented, with limited reports on its physiological functions, substrate spectrum, and transport characteristics (Amini et al. 2022; Stanton et al. 2022). The Arabidopsis ZIP family has 15 members, namely AtZIP1-AtZIP12 and AtIRT1-3. Ten (ZIP1-4, 6, 7,9,11–12 and IRT3) of them have been shown to complement the Zn-deficiency growth phenotype of the yeast Zn uptake defect mutant *zrt1zrt2* (Grotz et al. 1998; Lin et al. 2009; Assunção et al. 2010; Milner et al. 2013; Ajeesh Krishna et al. 2020; Lee et al. 2021). IRT1 and IRT2 have been well studied, but they mainly transport Fe^2+^ (Stanton et al. 2022). ZIP4/6/9 and IRT3 play an important role in the process of Zn loading in xylem, and the Zn content in shoots and seeds of *zip4/6/9irt3* high-order mutants was significantly reduced (Lee et al. 2021). ZIP2, located in the plasma membrane and predominantly expressed in roots, is implicated in Zn and Mn transport between pericycle cells in the root. Notably, the *zip2* knockout mutant exhibits tolerance to Zn deficiency but sensitivity to high Zn stress, and genetic evidence suggests that AtZIP2 plays a pivotal role in the distribution of Zn^2+^ from roots to shoots (Milner et al. 2013).

Calcium (Ca^2+^) serves as an omnipresent second messenger indispensable for plant growth and development, both under normal and stressful conditions (Wang and Li 2023; Zhang et al. 2025). In plants, the CBL and CIPK complex constitutes a critical component of Ca^2+^ signal decoding machinery, tasked with interpreting Ca^2+^ signals evoked by environmental fluctuations. To sustain nutrient homeostasis within plant cells, the CBL-CIPK module orchestrates the influx and efflux of nutrient ions by modulating the activity of ion transporters and channels (Verma et al. 2021). The CBL1/9-CIPK23 complex phosphorylates multiple channel proteins to regulate plant uptake of K^+^, NO_3_^−^, NH_4_^+^, Fe^2+^, Mn^2+^ and other nutrient ions (Xu et al. 2006; Ho et al. 2009; Maierhofer et al. 2014; Ragel et al. 2015; Straub et al. 2017; Zhang et al. 2023). CBL2/3-CIPK3/9/23/26 complex regulates intracellular Mg^2+^, K^+^ and Mn^2+^ homeostasis (Tang et al. 2015, 2020; Zhang et al. 2021; Ju et al. 2022). Our most recently study found that CBL1/4/5/8/9-CIPK3/9/23/26, through the perception of specific Ca^2+^ signals, mediates the phosphorylation of ZIP12, thereby regulating its protein degradation and playing a pivotal role in maintaining Zn homeostasis in plants (Fang et al. 2025). In essence, the CBL-CIPK complex functions as a specialized Ca^2+^ sensor and 'decoder' in plants, regulating the absorption and transport of nutrients within the plant.

In this study, we found that CBL1/4/5/8/9-CIPK3/9/23/26 interacts with and phosphorylates the plasma membrane-localized Zn transporter ZIP2, activating ZIP2-mediated Zn uptake into root stellar cells. This process serves as an essential prerequisite for the mobilization of Zn within the xylem parenchyma, followed by their compartmentalization into the xylem vessels and subsequent long-distance translocation to the stem through the transpiration stream. Notably, the hybrid complementation lines carrying CBL-CIPK-mediated phosphorylation sites of ZIP2 and ZIP12 exhibited enhanced tolerance to Zn deficiency, further validating the critical role of CIPK3/9/23/26-mediated phosphorylation of ZIP2 and ZIP12 in improving plant tolerance to Zn deficiency. In summary, we discovered that CBL-CIPK-ZIP2/ZIP12 phosphorylation network coordinates Zn allocation in Arabidopsis, offering valuable genetic resources for future molecular breeding efforts.

## Results

### CIPK3/9/23/26 interacts with Zn transporter ZIP2

**Fig. 1:**
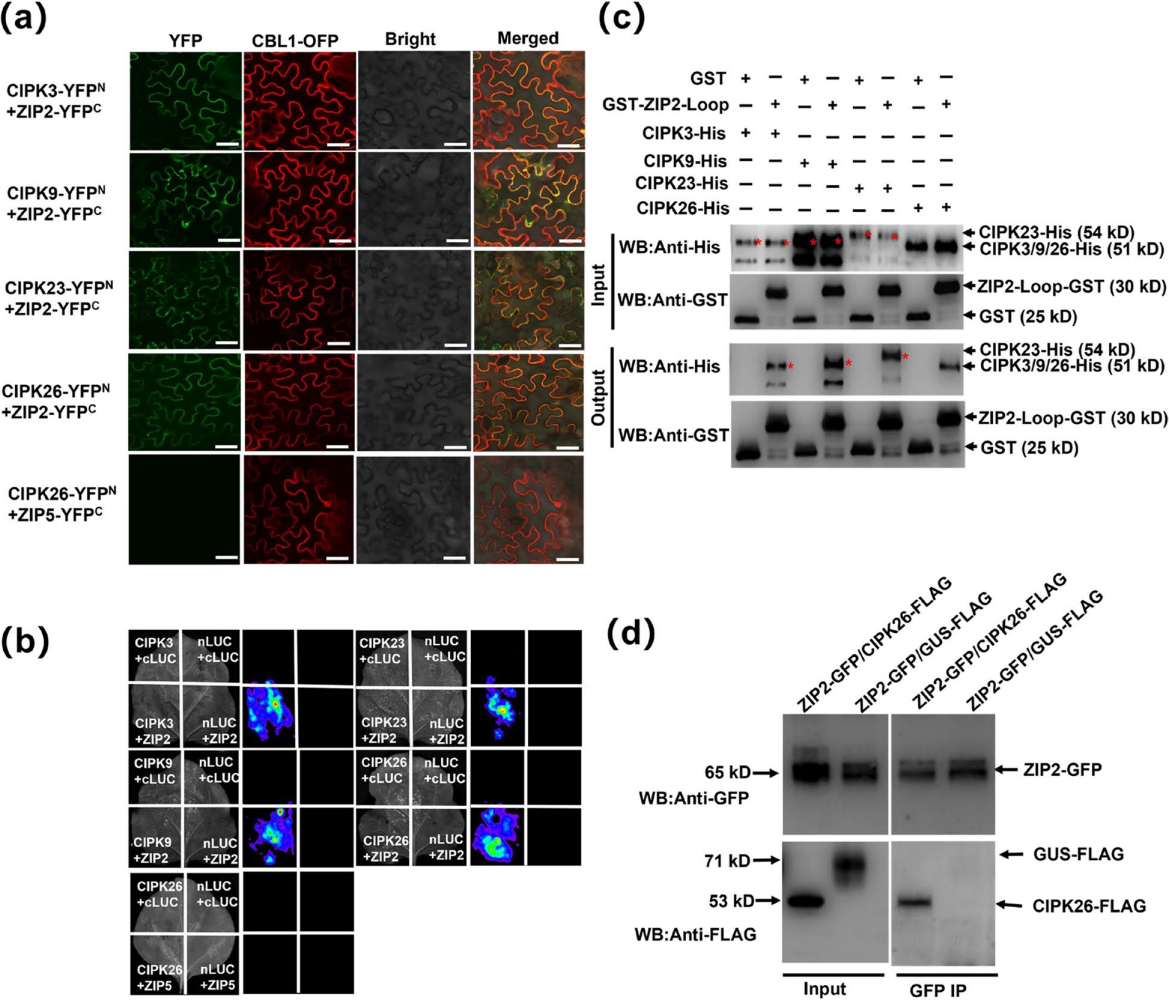
CIPK3/9/23/26 physically interacts with ZIP2. **a** CIPK3/9/23/26-nYFP + ZIP2-cYFP and CIPK26-nYFP + ZIP5-cYFP vectors were constructed and co-expressed with CBL1-OFP (a plasma membrane marker) in *Nicotiana benthamiana*. Bars, 40 μm. **b** LCI assay showing the interaction between CIPK3/9/23/26-nLUC and ZIP2-Cluc. CIPK26-nLUC and ZIP5-cLUC, ZIP2-cLUC/GUS-nLUC, GUS-cLUC/CIPK3/9/23/26-nLUC, and GUS-cLUC/GUS-nLUC were used as negative controls. **c** Pull-down assay showing the interaction between CIPK3/9/23/26 and ZIP2. CIPK3/9/23/26 to His and ZIP2-Loop was fused to GST. The input and output were analyzed via western-blotting with anti-His and anti-GST antibodies. Input, the sample taken after incubation of two proteins; Output, protein samples eluted from beads. The asterisk indicates the target protein. **d** Co-immunoprecipitation of CIPK26 with ZIP2 in CIPK26-FLAG/ZIP2-GFP Arabidopsis transgenic plants. GUS-FLAG/ZIP2-GFP transgenic plants were used as negative controls. Co-IP, co-immunoprecipitation; Input, positive control; IP, Immunoprecipitation

In previous studies, we observed the occurrence of fluorescence upon co-injecting CIPK26 and ZIP2 in tobacco plants, during the analysis of interactions between 11 members of the ZIP family, localized to the plasma membrane, and CIPK26 through the application of bimolecular fluorescence complementation (BiFC) (Fang et al. 2025). To delve deeper into the mechanisms by which CIPK3/9/23/26 and ZIP2 regulate Zn transport in Arabidopsis, we initially employed BiFC and split luciferase complementation imaging (LCI) techniques. Specifically, for the BiFC assay, we utilized the plasma membrane marker CBL1-OFP. Our results revealed robust fluorescence signals on the plasma membrane upon co-expression of CIPK3/9/23/26-nYFP and ZIP2-cYFP. Conversely, the negative controls, comprising combinations such as CIPK26-nYFP + ZIP5-cYFP, CIPK3/9/23/26-nYFP + GUS-cYFP and GUS-nYFP + ZIP2-cYFP did not yield any fluorescence signals. These findings indicate a specific interaction between CIPK3/9/23/26 and ZIP2 (Fig. 1a, Fig. S1a and b). Furthermore, the outcomes of LCI assays corroborated these interactions, demonstrating intense fluorescence upon co-injection of CIPK3/9/23/26-nLUC and ZIP2-cLUC. No fluorescence was detected in the co-injection of negative control pairs, namely CIPK26-nLUC + ZIP5-cLUC, GUS-nLUC + ZIP2-cLUC, GUS-cLUC + CIPK3/9/23/26-nLUC, and GUS-nLUC + GUS-cLUC (Fig. 1b and Fig. S1c). These observations provide additional support for the existence of a protein–protein interaction between CIPK3/9/23/26 and ZIP2.

Subsequently, we conducted an investigation into the interaction between CIPK3/9/23/26 and ZIP2 in vitro, employing Glutathione S-transferase (GST) pull-down assays. Given the complexity associated with purifying the full-length ZIP2 protein, which contains nine transmembrane regions, we directed our focus towards the largest Loop domain (151–203 aa) within ZIP2. The GST pull-down assays demonstrated that CIPK3/9/23/26-His proteins exhibited specific interactions with GST-ZIP2-Loop in vitro, whereas no interaction was observed with GST alone (Fig. 1c).

To further explore the in vivo interaction between CIPK3/9/23/26 and ZIP2, we conducted co immunoprecipitation (Co-IP) experiments in transgenic Arabidopsis plants expressing CIPK26-FLAG/ZIP2-GFP and GUS-FLAG/ZIP2-GFP constructs. In the Co-IP experiments, precipitated CIPK26 proteins were identified using an anti-GFP antibody. We found that CIPK26 selectively interacted with ZIP2 in transgenic plants overexpressing CIPK26-FLAG/ZIP2-GFP, whereas CIPK26 was undetectable in the immunoprecipitation (IP) samples from transgenic plants expressing GUS-FLAG/ZIP2-GFP (Fig. 1d). This indicates that CIPK26 binds specifically to ZIP2. Cumulatively, these findings underscore and affirm the binding interaction of CIPK3/9/23/26 with ZIP2 both in vivo and in vitro.

### CBL1/4/5/8/9-CIPK3/9/23/26 acts upstream of ZIP2

We subsequently investigated the genetic interactions between CBL1/4/5/8/9-CIPK3/9/23/26 and ZIP2 in Arabidopsis. Prior studies have demonstrated that higher-order mutants of *cbl1/4/5/8/9*, *cipk3/9/23/26*, and single mutants of *zip2* exhibit tolerance to Zn deficiency (Milner et al. 2013; Fang et al. 2025). To further elucidate these interactions, we generated Arabidopsis plants overexpressing CIPK26 (CIPK26OE) and subjected them to Zn deficiency conditions. However, no significant phenotypic differences were observed between CIPK26OE and WT plants (Fig. S2a-c). This prompted speculation that CIPK26's response to Zn deficiency might be influenced by factors beyond its expression level, potentially including its kinase activity. To test this hypothesis, we constructed constitutively active CIPK26 overexpression plants (CIPK26^T172D^OE) (Guo et al. 2001). Intriguingly, CIPK26^T172D^OE overexpressing plants exhibited sensitivity to Zn deficiency, with significantly reduced root length compared to WT plants (Fig. S2d-e).

**Fig. 2:**
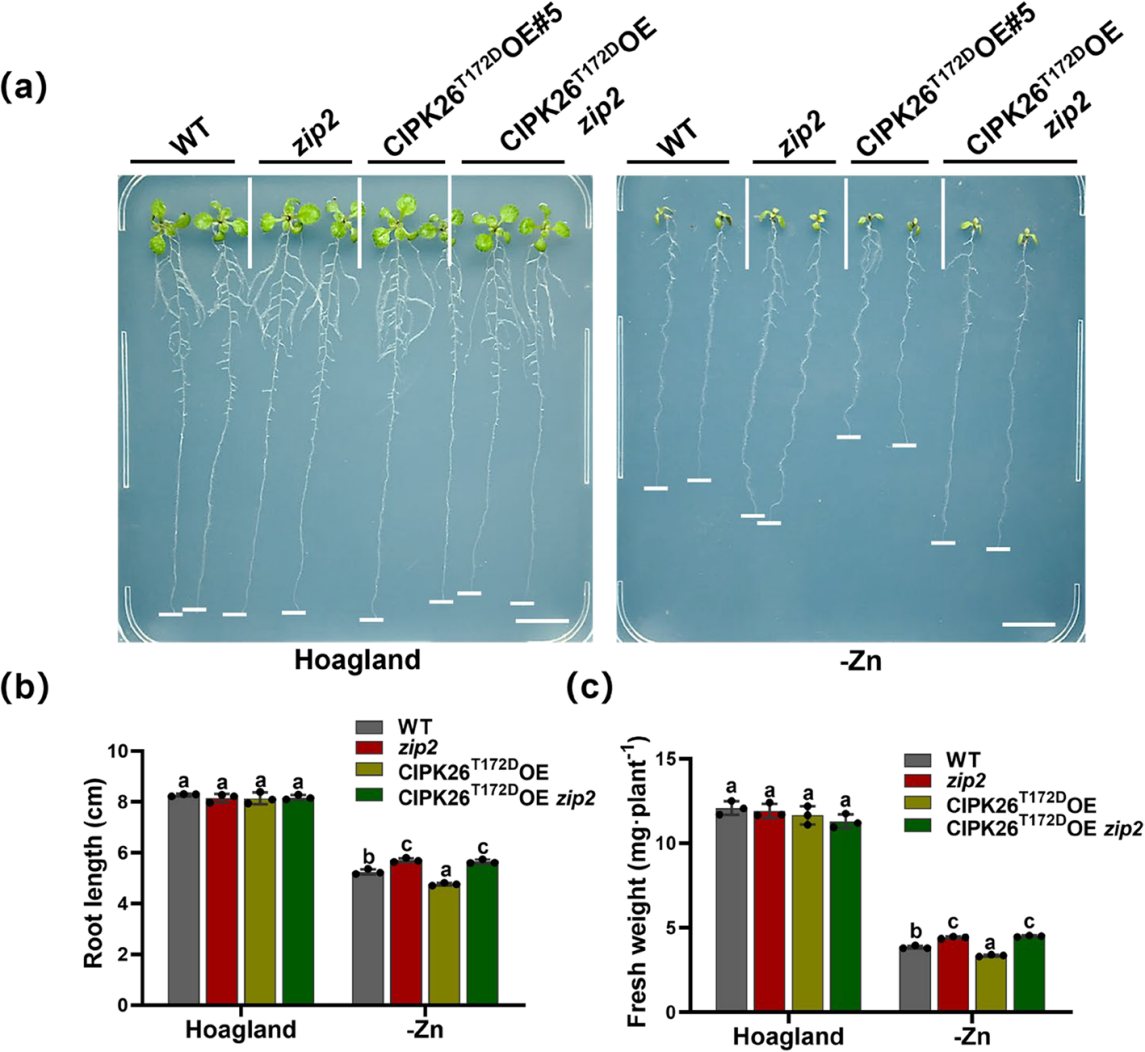
Genetic interaction of CIPK3/9/23/26 and ZIP2 in Arabidopsis. **a** Zn-deficiency phenotype of CIPK26^T172D^OE/zip2. Scale bars, 1 cm. **b** Statistical analysis of primary root lengths of plants shown in (**a**). The data are presented as the mean ± SD (*n* = 16 seedlings for each genotype). **c** Statistics on the fresh weight of the plants in (**a**). The data are presented as the mean ± SD (*n* = 16 seedlings for each genotype). Statistical differences were calculated by one-way ANOVA. Different letters indicate means that were statistically different by Tukey's multiple testing method (*P* < 0.05) for genotypes within a given growth condition (Hoagland or -Zn)

To further investigate the genetic interactions, we crossed CIPK26^T172D^OE overexpressing plants with *zip2* mutants and analyzed the resulting phenotype under Zn deficiency conditions. Our results revealed that CIPK26^T172D^OE plants displayed a Zn deficiency-sensitive phenotype, whereas CIPK26^T172D^OE*zip2* exhibited the same Zn deficiency tolerance phenotype as the *zip2* mutant, characterized by increased main root length (Fig. 2a-c). These findings suggest that CBL1/4/5/8/9-CIPK3/9/23/26 likely functions upstream of ZIP2 in the Zn deficiency response pathway in Arabidopsis.

### CIPK3/9/23/26 phosphorylates ZIP2 primarily at Ser190

**Fig. 3:**
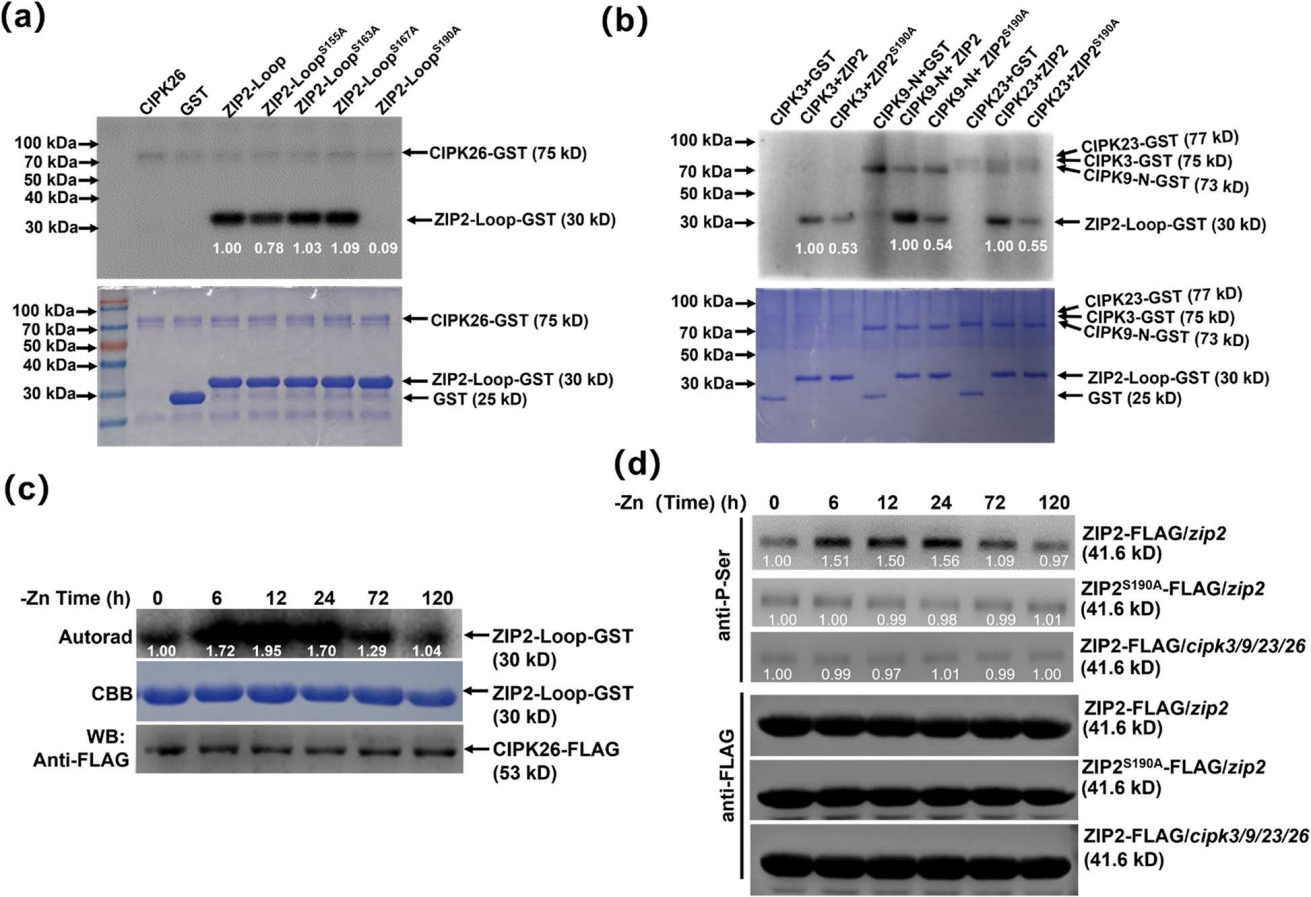
CIPK3/9/23/26 phosphorylates ZIP2 at Ser190. **a** Ser190 was essential for the phosphorylation of ZIP2 by CIPK26. Recombinant purified GST-CIPK26 was incubated with GST-ZIP2-Loop, its mutant forms, or GST in kinase reaction buffer with 1 μCi (γ-^32^P) ATP for 30 min at 30 °C. The proteins were separated by SDS-PAGE. Top, autoradiograph; bottom, CBB staining. **b** Ser190 was essential for the phosphorylation of ZIP2 by CIPK3/9/23. The proteins were separated by SDS-PAGE. Top, autoradiograph; bottom, CBB staining. **c** Protein kinase assay of CIPK26 with ZIP2-Loop under Zn-deficiency stress. 7-day-old Arabidopsis seedlings were treated with a Zn-deficiency medium for the indicated time periods. The protein kinases were quantified by SDS-PAGE and are shown at the bottom. **d** The seedlings were treated with Zn-deficiency conditions for 0, 6, 12, 24, 72 and 120 h, respectively. The phosphorylation signals of ZIP2 were detected by anti-P-Ser antibodies and anti-FLAG antibody

Previous studies have documented that CIPK functions as a protein kinase-mediated downstream target protein phosphorylation event (Luan 2009; Ju et al. 2022). To investigate whether CIPK3/9/23/26 directly phosphorylates ZIP2-Loop, we performed in vitro kinase assays using purified CIPK3/9/23/26-GST and ZIP2-Loop-GST. The results showed that CIPK3/9/23/26 could phosphorylate the Loop domain of ZIP2 (Fig. 3a). To further elucidate the primary phosphorylation sites within ZIP2, we identified four potential phosphorylation sites (Ser155, Ser163, Ser167, and Ser190) in the ZIP2-Loop domain, based on predictions from a phosphorylation prediction website (http://gps.biocuckoo.org/). We subsequently mutated these four serine residues to glycine and fused the mutated proteins with GST tags, enabling the construction and purification of four mutant proteins: ZIP2-Loop^S155A^-GST, ZIP2-Loop^S163A^-GST, ZIP2-Loop^S167A^-GST and ZIP2-Loop^S190A^-GST. To pinpoint the phosphorylation sites on ZIP2-Loop targeted by CIPK26, we conducted additional in vitro kinase assays. Notably, the phosphorylation levels of ZIP2-Loop^S155A^ and, particularly, ZIP2-Loop^S190A^ were markedly reduced compared to ZIP2-Loop (Fig. 3a). Furthermore, the phosphorylation of ZIP2-Loop^S190A^ by the other three protein kinases, CIPK3, CIPK9, and CIPK23, was also significantly diminished (Fig. 3b). It is worth mentioning that, by utilizing the phosphorylation mass spectrometry database (http://phosphat.uni-hohenheim.de/), it was discovered that Ser190 can also be phosphorylated in vivo. This indicates that CIPK3/9/23/26 mainly phosphorylates the Ser190 site of ZIP2.

To elucidate the potential regulatory role of phosphorylation on ZIP2 in the plant's response to Zn deficiency, we investigated whether Zn deficiency alters the phosphorylation level of ZIP2. For this purpose, transgenic plants overexpressing FLAG-tagged CIPK26 were subjected to Zn-deficiency conditions for durations of 0, 6, 12, 24, 72, and 120 h. Following these treatments, the CIPK26-FLAG protein was extracted and incubated with the prokaryotic fusion protein ZIP2-Loop-GST in the presence of 1 μCi [γ-^32^P] ATP. Our observations revealed a significant increase in the phosphorylation level of ZIP2 mediated by CIPK26 from 6 to 24 h, followed by a gradual decline (Fig. 3c). The finding suggested that ZIP2 phosphorylation by CIPK26 is inducible by Zn deficiency.

In order to further mechanically link the Zn deficiency tolerance phenotype of *cbl1/4/5/8/9* and *cipk3/9/23/26* high-order mutants with CIPK3/9/23/26 activation-dependent Ser190 phosphorylation status, we performed time series analysis of ZIP2 phosphorylation in Zn deficiency response. Therefore, ZIP2-FLAG/*zip2*, ZIP2^S190A^-FLAG/*zip2* and ZIP2-FLAG/*cipk3/9/23/26* transgenic plants were treated with Zn deficiency for 0, 6,12, 24, 72 and 120 h, respectively. ZIP2 was extracted from these plants, enriched with anti-GFP antibody, and the phosphorylation level of ZIP2 was detected with anti-P-Ser antibody. Using anti-P-Ser antibody, compared with ZIP2-FLAG/*zip2* transgenic plants, the phosphorylation level of ZIP2 in ZIP2^S190A^-FLAG/*zip2* and ZIP2-FLAG/*cipk3/9/23/26* transgenic plants was significantly reduced. In addition, we observed that the phosphorylation of ZIP2 was also significantly enhanced at 6 h of Zn deficiency, which was consistent with the result that the kinase activity of CIPK26 began to activate at 6 h of Zn deficiency. In contrast, the phosphorylation level of ZIP2 in ZIP2^S190A^-FLAG/*zip2* and ZIP2-FLAG/*cipk3/9/23/26* transgenic plants were almost unchanged (Fig. 3d). Taken together, these results clearly indicate that Zn deficiency-induced phosphorylation of Ser190 in ZIP2 is strictly dependent on the activity of CIPK3/9/23/26.

### ZIP2^Ser190^ phosphorylation is crucial for ZIP2 transport activity

**Fig. 4:**
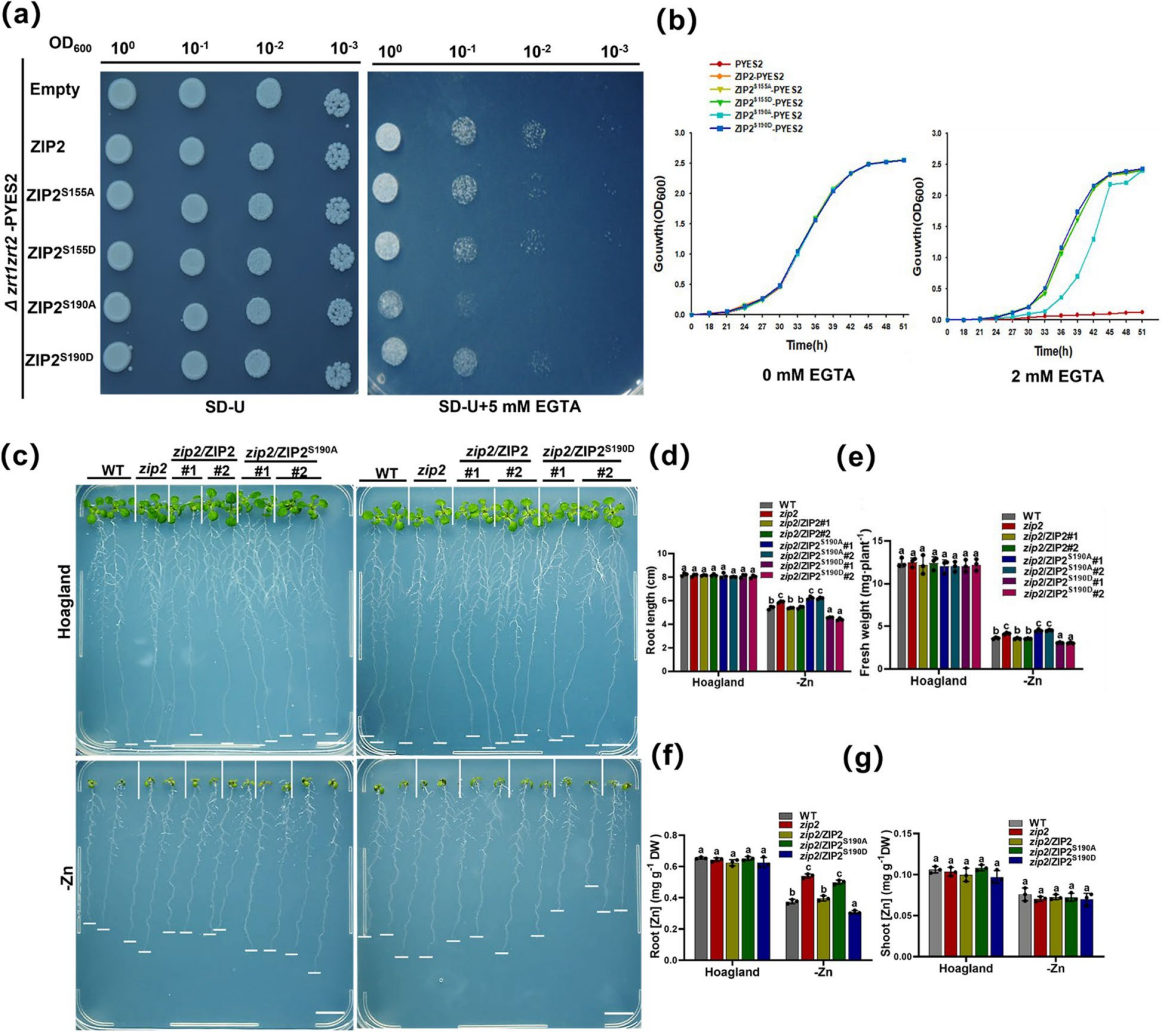
Functional analysis of ZIP2 phosphorylated by CIPK3/9/23/26. **a** Empty vector (Δ*zrt1zrt2* + pYES2), ZIP2 (Δ*zrt1zrt2* + ZIP2), and ZIP2 variants (Δ*zrt1zrt2* + ZIP2 variants) were transferred into the yeast mutant strain Δzrt1zrt2. Yeast cells were grown on normal medium (SD-U, lacking Ura) and treatment medium (SD-U + 5 mM EGTA) for 3–5 d. Four tenfold series of diluents were established under sterile conditions. **b** Growth curves of yeast cells expressing pYES2, ZIP2, and ZIP2 variants were plotted from OD_600_ values. Growth of yeast cells in liquid cultures containing 0 or 2 mM EGTA was monitored every 3 h from 0 to 51 h (*n* = 3 biological replicates). **c **The Zn deficiency phenotype of ProZIP2:ZIP2, ProZIP2:ZIP2^S190A^ and ProZIP2:ZIP2^S190D^ transgenic plants. Plants were grown under Hoagland and -Zn conditions for 10 days. #1 and #2 are independent transgenic lines. Scale bars, 1 cm. **d** The column shows the statistics of the primary root length of the plants in (**c**). **e** Statistics on the fresh weight (FW) of the plants in (**c**). The data are presented as the mean ± SD (*n* = 16 seedlings for each genotype). **f** Analysis of Zn content in plant roots in (**c**). **g** Analysis of Zn content in plant shoots in (**c**). The data are the mean ± SD (*n* = 3 biological replicates). Statistical differences were calculated by one-way ANOVA. Different letters indicate means that were statistically different by Tukey’s multiple testing method (*P* < 0.05) for genotypes within a given growth condition (Hoagland or -Zn)

To investigate the biological significance of Ser190 phosphorylation in ZIP2, we initiated our study by conducting heterologous yeast complementation experiments. Previous research has demonstrated that Arabidopsis ZIP2 can complement the Zn-sensitive phenotype of the yeast Zn uptake-defictive strain *zrt1zrt2* (Milner et al. 2013). Subsequently, we constructed ZIP2 variants, including a simulated non-phosphorylated form (ZIP2^S155A^, ZIP2^S190A^), and phosphorylated mimics (ZIP2^S155D^, ZIP2^S190D^), and cloned them into the vector PYES2. These constructs were then transformed into the Zn uptake-defictive yeast strain *zrt1zrt2* for expression (Zhao and Eide 1996). The experiments were carried out according to established methodologies described in prior publications (Lee et al. 2021). There was no significant difference in the growth of yeast on SD/-Ura medium. However, when 5 mM EGTA was introduced into the medium to chelate Zn^2+^, resulting in Zn depletion, significant differences in growth ability among the yeast strains became evident. The results indicated that yeast grown in Zn-deficiency medium exhibited substantial growth impairment compared to those grown on SD/-Ura medium. Notably, *zrt1zrt2* yeast harboring only the empty vector PYES2 significantly inhibited growth under Zn deficiency conditions. Furthermore, yeast expressing the non-phosphorylated ZIP2^S190A^ variant displayed a marked reduction in growth ability (Fig. 4a).

We conducted an analysis of the growth curves of *Δzrt1zrt2* yeast cultures in media supplemented with or without 2 mM EGTA. Our experimental results revealed that, under Zn deficiency conditions, the growth rates of *Δzrt1zrt2* yeast carrying either the empty vector or the simulated non-phosphorylated ZIP2^S190A^ variant were substantially diminished. Conversely, the growth rates of yeast expressing all other ZIP2 constructs, including ZIP2 and its phosphorylated variants, remained comparable under Zn deficiency (Fig. 4b). These findings suggest that phosphorylation at Ser190 is crucial for the Zn transport function of ZIP2, whereas the phosphorylation status of Ser155 does not appear to play a regulatory role in its transport activity.

We further examined the impact of the phosphorylation status of Ser190 on the subcellular localization of ZIP2. To this end, transgenic plants expressing ZIP2 fused with green fluorescent protein (GFP), as well as ZIP2 variants with non-phosphorylatable (ZIP2^S190A^-GFP) and constitutively phosphorylated (ZIP2^S190D^-GFP) Ser190 residues, were generated and cultivated for four days in both Hoagland medium and Zn-deficiency medium. Subsequently, confocal microscopy was employed to analyze their fluorescence patterns. Our findings demonstrate that the subcellular localization of ZIP2^S190A^-GFP and ZIP2^S190D^-GFP did not deviate from that of ZIP2-GFP in either Hoagland medium or Zn-deficiency medium (Fig. S3). These results indicate that the phosphorylation status of Ser190 does not exert an influence on the subcellular localization of ZIP2.

To ascertain whether the phosphorylation status of the Ser190 residue impacts the protein accumulation of ZIP2, we constructed ZIP2-FLAG and ZIP2^S190A^-FLAG vectors and successfully transformed them into *zip2* mutant plants, yielding two transgenic lines. Subsequently, we conducted immunoblotting analysis using an anti-FLAG antibody to assess the accumulation of ZIP2 protein in these lines. The transgenic plants were initially cultivated in Hoagland medium for seven days before being transferred to either Zn-deficiency medium or Zn-deficiency medium supplemented with 10 μM MG132 or cycloheximide (CHX). Samples were collected at 0, 6, 12, 24, 72, and 120 h after treatment. Protein levels of ZIP2 and ZIP2^S190A^ were assessed throughout the time course. No significant differences in protein accumulation were observed between ZIP2 and ZIP2^S190A^ (Fig. S4a–c). These findings suggest that the phosphorylation state of Ser190 does not influence the accumulation levels of ZIP2 protein.

Subsequently, we aimed to elucidate the physiological significance of Ser190 phosphorylation. To achieve this, we generated transgenic plants expressing ZIP2, ZIP2^S190A^, and ZIP2^S190D^ variants under the control of the ZIP2 promoter in a ZIP2 mutant background (Fig. S5a). These transgenic lines, along with WT plants and *zip2* mutants, were cultivated in both Hoagland medium and Zn-deficiency medium for ten days. Notably, the growth phenotype of ProZIP2:ZIP2 transgenic plants in Zn-deficiency conditions resembled that of the WT plants. It is pertinent to mention that, in terms of root length and fresh weight, ProZIP2:ZIP2^S190A^ transgenic plants exhibited a phenotype akin to the Zn deficiency tolerance observed in *zip2* mutants. Conversely, ProZIP2:ZIP2^S190D^ transgenic lines displayed a Zn deficiency-sensitive phenotype (Fig. 4c-e).

To further ascertain whether the Zn deficiency tolerance phenotype observed in ProZIP2:ZIP2^S190A^ transgenic plants and the Zn deficiency sensitivity phenotype in ProZIP2:ZIP2^S190D^ transgenic lines were attributed to variations in Zn concentration within the plants, we conducted a quantitative analysis of Zn concentration in the roots and shoots of WT plants, *zip2* mutants, and transgenic lines expressing ProZIP2:ZIP2, ProZIP2:ZIP2^S190A^, and ProZIP2:ZIP2^S190D^. The findings revealed that under Zn-deficiency conditions, the Zn concentration in the roots of *zip2* mutants and ProZIP2:ZIP2^S190A^ transgenic plants was significantly elevated compared to that in WT and ProZIP2:ZIP2 plants. However, no discernible difference was observed in Zn concentration within the shoots among these genotypes. Conversely, the Zn concentration in the roots of ProZIP2:ZIP2^S190D^ transgenic lines was markedly decreased compared to that in WT and ProZIP2:ZIP2 plants under Zn-deficiency conditions, with no significant difference noted in Zn concentration within the shoots. These results suggest that the altered Zn accumulation patterns in the roots of these transgenic lines are likely responsible for their respective Zn deficiency tolerance and sensitivity phenotypes (Fig. 4f and g).

We further characterized the concentrations of Fe, Mn, and Cu in these plant samples. Notably, a significant reduction in Mn concentrations was specifically observed in the roots of *zip2* mutants and ProZIP2:ZIP2^S190A^ transgenic plants. Prior studies in yeast and plants have provided preliminary insights into the ZIP2 transporter, suggesting its potential role in facilitating the transport of both Zn and Mn in roots (Milner et al. 2013). Given these findings, we postulate that the alterations in Mn content observed in the roots of *zip2* mutants and ProZIP2:ZIP2^S190A^ transgenic plants under Zn-deficiency conditions may be attributed to the involvement of ZIP2 in Mn transport processes. No significant differences were observed in the concentrations of the other metals analyzed (Fig. S6). Taken together, the phenotypic observations and elemental content analysis presented here underscore the pivotal role of Ser190 phosphorylation in ZIP2-mediated Zn transport into the root vasculature, which is crucial for subsequent translocation to the shoots.

### CIPK3/9/23/26-mediated phosphorylation of Zn transporters ZIP12 and ZIP2 enhances plant tolerance to Zn deficiency stress

**Fig. 5:**
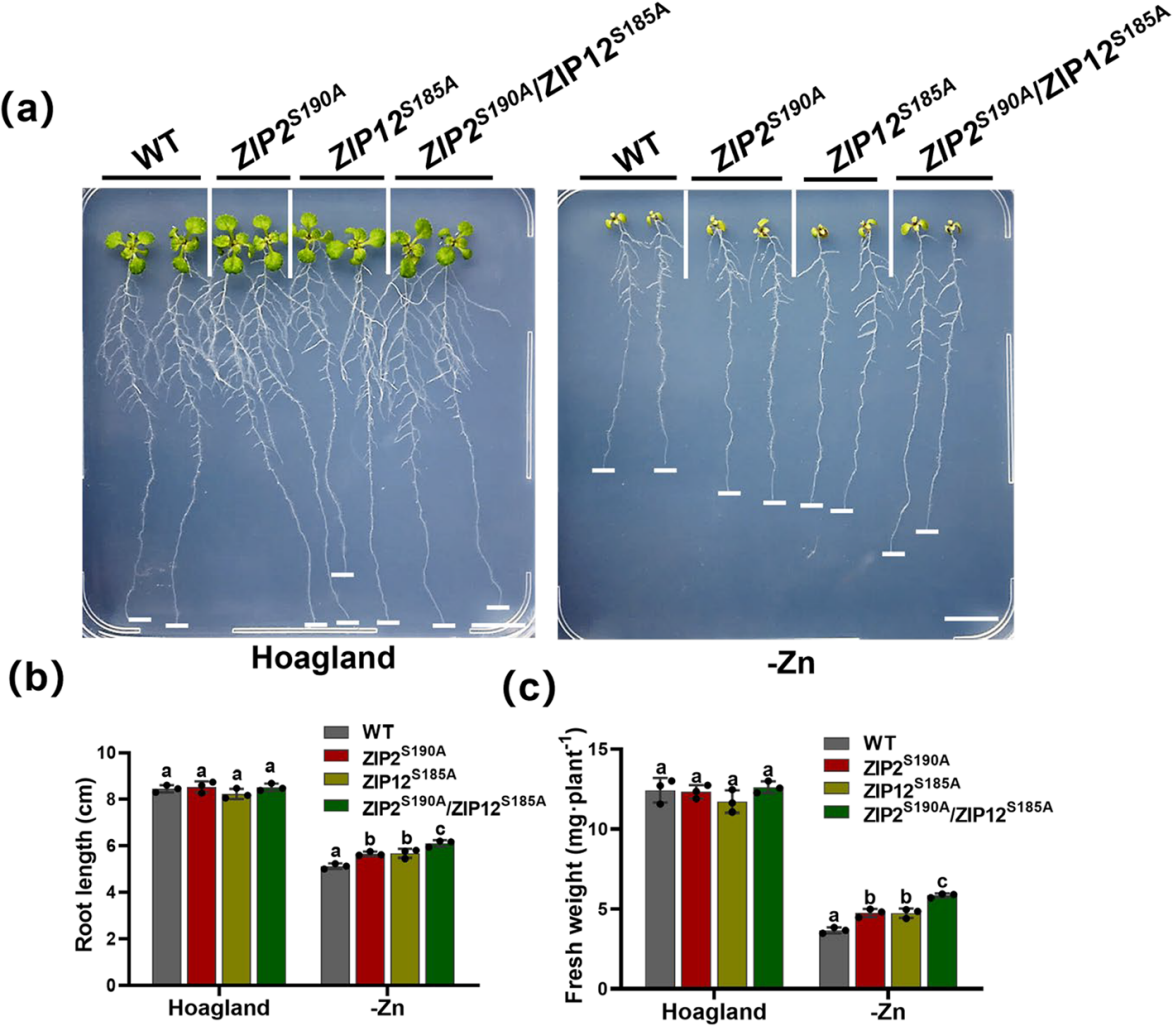
Phenotypic analysis of Zn deficiency in CIPK3/9/23/26-mediated ZIP2 and ZIP12 phosphorylation site transgenic lines. **a **The Zn deficiency phenotype of *zip2/ZIP2*^*S190A*^*, zip12/ZIP12*^*S185A*^* and ZIP2*^*S190A*^*/ZIP12*^*S185A*^ transgenic plants. These plants were grown on Hoagland conditions and -Zn conditions. Scale bars, 1 cm. **b** Statistical analysis of root lengths of plants shown in (**a**). **c** Statistical analysis of fresh weight of plants shown in (**a**). The data are presented as the mean ± SD (n = 16 seedlings for each genotype). Statistical differences were calculated by one-way ANOVA. Different letters indicate means that were statistically different by Tukey’s multiple testing method (*P* < 0.05) for genotypes within a given growth condition (Hoagland or -Zn)

Our recent research has revealed that under conditions of Zn deficiency, the kinases CIPK3/9/23/26 predominantly facilitate the degradation of the ZIP12 protein via phosphorylation at the Ser185 residue (Fang et al. 2025). Additionally, our investigation demonstrates that CIPK3/9/23/26 orchestrate Zn transport in roots through phosphorylation of the Ser190 residue of ZIP2. Phenotypic analysis of Zn deficiency indicated that transgenic plants harboring mutations at the Ser185 site of ZIP12 (*zip12*/ZIP12^S185A^) and the Ser190 site of ZIP2 (*zip2*/ZIP2^S190A^) exhibited enhanced tolerance to Zn deficiency. Consequently, we formulated the hypothesis that CIPK3/9/23/26-mediated phosphorylation of the Zn transporters ZIP2 and ZIP12 could further augment plant resilience to Zn deficiency stress. To test this hypothesis, we conducted genetic crosses between *zip12*/ZIP12^S185A^ and *zip2*/ZIP2^S190A^ transgenic plants and evaluated the Zn deficiency phenotype of the resultant ZIP2^S190A^/ZIP12^S185A^ plants (Fig. S5b and c). Our results indicate that ZIP2^S190A^/ZIP12^S185A^ plants displayed greater Zn deficiency tolerance compared to *zip12*/ZIP12^S185A^ and *zip2*/ZIP2^S190A^ plants, evidenced by significant increases in main root length and fresh weight (Fig. 5a-c). These results suggest that the CIPK3/9/23/26-mediated phosphorylation of Zn transporters ZIP2 and ZIP12 can indeed enhance plant tolerance to Zn deficiency stress, offering valuable insights for the development of trace element hyperaccumulator crops.

## Discussion

Nutrient deprivation poses a significant challenge to cellular function, eliciting intricate intracellular Ca^2+^ signaling dynamics. These fluctuations activate specific Ca^2+^ sensors, known as CBLs, and their cognate kinases, CIPKs, forming the CBL-CIPK signaling module. Upon activation, this module phosphorylates downstream targets, which often include ion channels and transporters, thereby maintaining cellular ion homeostasis (Luan 2009; Verma et al. 2021; Dong et al. 2022; Wang et al. 2023). In our most recently study and this study, we discovered that CBL1/4/5/8/9-CIPK3/9/23/26 mediates the absorption and translocation of Zn^2+^ in plant roots through the phosphorylation modification of Zn transporters ZIP2 and ZIP12. Higher-order mutants *cbl1/4/5/8/9* and *cipk3/9/23/26* exhibit significant tolerance phenotypes under Zn deficiency stress (Fang et al. 2025). However, plants overexpressing CIPK26 did not show obvious Zn deficiency phenotypes, whereas transgenic CIPK26^T172D^OE plants, which overexpress CIPK26 with a constitutively active kinase form, displayed Zn deficiency-sensitive phenotypes (Fig. S2). This suggests that the kinase activity of CIPK3/9/23/26 is primarily responsive to Zn deficiency.

In contrast to the canonical phosphorylation-mediated mechanisms involving protein degradation or subcellular relocalization (Boudsocq and Sheen 2013; Yang and Guo 2018; Chen et al. 2021; Ju et al. 2022). The Zn transporter ZIP2, which is homologous to ZIP12, exhibits fundamentally distinct biological functions under regulation by the identical CBL-CIPK kinase module: A targeted screen of plasma membrane-localized Zn transporters identified a specific interaction between CIPK26 and ZIP2. Subsequent in *vivo* and in *vitro* experiments confirmed that CIPK3/9/23/26 phosphorylate Ser190 of ZIP2, activating its Zn^2+^ transport activity without promoting degradation. This positive regulatory mechanism drives directional Zn^2+^ influx into root stellar cells, serving as a rate-limiting step for Zn activation in xylem parenchyma cells and subsequent compartmentalization into xylem vessels. Crucially, ZIP2 function displays marked spatial specificity—its high expression in root vascular tissues and phosphorylation-dependent activation complements ZIP12-mediated Zn uptake in root epidermis (Milner et al. 2013; Fig. 3), collectively establishing a dual-module "absorption-translocation" regulatory network for plant adaptation to Zn deficiency. Our findings not only uncover a novel mechanism whereby the CBL-CIPK module achieves spatiotemporal Zn allocation through differential regulation of homologous transporters, but also provide ZIP2 as a precision-breeding target for developing Zn-efficient crops.

The Zn transporters ZIP12 and ZIP2 have distinct functions in plants. ZIP12 is primarily responsible for absorbing Zn from the soil into the plant roots, while based on the high expression of ZIP2 in the stele and the observed phenotypes of T-DNA ZIP2 knockout lines, ZIP2 is likely involved in mediating Zn transport into the root vasculature for translocation to the shoot, rather than absorbing Zn from the soil (Milner et al. 2013; Inaba et al. 2015). Interestingly, we found that CBL1/4/5/8/9-CIPK3/9/23/26 can regulate the protein accumulation of ZIP12 through phosphorylation and also phosphorylate ZIP2 to mediate Zn transport into root stellar cells, promoting Zn movement in the xylem parenchyma. Notably, the hybrid complementation lines carrying CBL-CIPK-mediated phosphorylation sites of ZIP2 and ZIP12 exhibited enhanced tolerance to Zn deficiency, further validating the critical role of CIPK3/9/23/26-mediated phosphorylation of ZIP2 and ZIP12 in improving plant tolerance to Zn deficiency (Fig. 5). Moreover, this genetic cross provides a more intuitive demonstration of the phenotypic effects conferred by distinct mutation sites, offering valuable genetic resources for future molecular breeding efforts.

## Conclusion

**Fig. 6:**
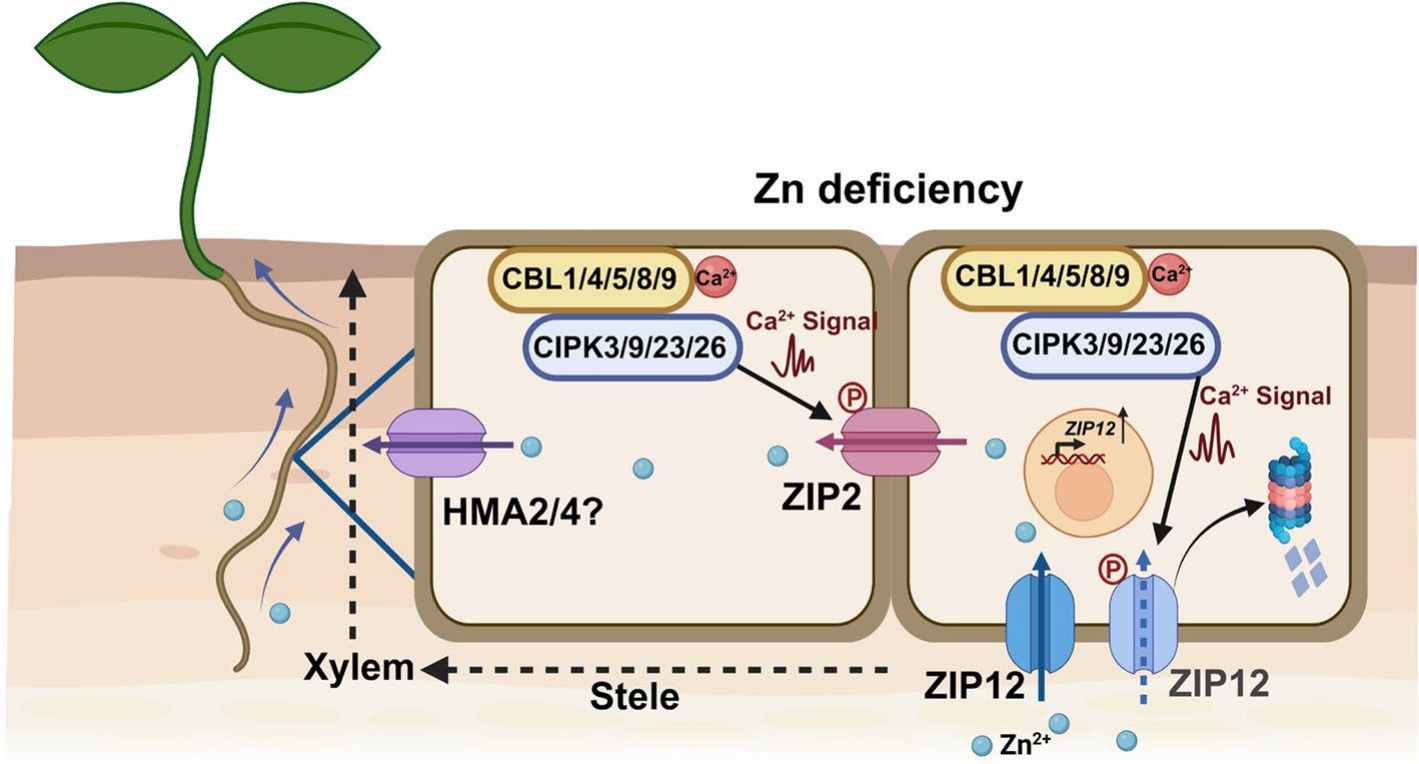
Model of the CBL-CIPK-ZIP2/ZIP12 phosphorylation network coordinates Zn allocation in Arabidopsis. This study reveals for the first time the molecular mechanism by which the CBL-CIPK module dynamically regulates the Zn transporters ZIP2 and ZIP12 through site-specific phosphorylation. Under Zn-deficiency conditions, the CBL1/4/5/8/9-CIPK3/9/23/26 complex activates the transport activity of ZIP2 via phosphorylation, mediating Zn uptake in root cortical cells and its directional transport to xylem parenchyma. Simultaneously, Zn loading into the xylem is likely coordinated through the Zn efflux transporters AtHMA2/4, ensuring efficient Zn transport to the shoot via the transpiration stream. Additionally, this regulatory network mediates partial degradation of ZIP12 through the ubiquitin–proteasome pathway via phosphorylation. This negative feedback mechanism not only prevents the excessive accumulation of Zn transporters due to plant stress responses but also dynamically adapts to fluctuations in environmental Zn concentrations, thereby maintaining physiological balance in Zn uptake by precisely regulating ZIP12 protein homeostasis

This study systematically deciphered the calcium signaling network regulating Zn homeostasis in plants, and for the first time elucidated the molecular mechanism by which the CBL-CIPK module dynamically regulates the Zn transporters ZIP2 and ZIP12 through site-specific phosphorylation. Under Zn deficiency conditions, the CBL1/4/5/8/9-CIPK3/9/23/26 complex activates the transport activity of ZIP2 via phosphorylation, mediating Zn uptake into root stellar cells and its directional transport to xylem parenchyma. Simultaneously, Zn loading into the xylem is likely coordinated through the Zn efflux transporters AtHMA2/4, ensuring efficient Zn transport to the shoot via the transpiration stream. Additionally, this regulatory network mediates partial degradation of ZIP12 through the ubiquitin–proteasome pathway via phosphorylation. This negative feedback mechanism not only prevents the excessive accumulation of Zn transporters due to plant stress responses but also dynamically adapts to fluctuations in environmental Zn concentrations, thereby maintaining physiological balance in Zn uptake by precisely regulating ZIP12 protein homeostasis (Fig. 6). The study further confirmed that the coordinated regulation of multiple phosphorylation sites on Zn transporters by the CBL-CIPK module significantly enhances plant adaptability to Zn deficiency. The revealed multi-target coordinated regulatory strategy provides an important theoretical basis and technical pathway for the genetic improvement of Zn nutrition efficiency in crops.

## Materials and methods

### Plant materials and growth conditions

The WT plants used in this study were Columbia (Col-0) background. The T-DNA insertion lines *zip2* (SALK_045578) were obtained from Nottingham Arabidopsis Stock Centre (NASC). The Arabidopsis seeds were grown on a nutrient medium consisting of 1% agar (Sigma-Aldrich, USA), 1% sucrose, and full strength Hoagland’s nutrient solution [5 mM KNO_3_, 5 mM Ca(NO_3_)_2_, 2 mM MgSO_4_, 1 mM NH_4_H_2_PO_4_, 20 μM MnSO_4_, 3 μM H_3_BO_3_, 1 mM (NH_4_)_6_Mo_7_O_24_, 0.4 μM ZnSO_4_, 0.2 μM CuSO_4_, 20 μM Fe (III)-EDTA, 50 μM EDTA-2Na] and − Zn medium (full strength Hoagland’s nutrient solution without ZnSO_4_), grown on vertical plates at 21 °C for 10 d under a 16 h light/8 h dark cycle (Gao et al. 2021). For soil culture, after germination, 7-d-old seedlings growing on Hoagland medium were transplanted to nutrient-rich soil (Pindstrup substrate, Denmark) and then grown under normal light conditions (100 μmolm^−2^ s^−1^) and a long-day cycle (16 h:8 h, light:darkness) at 22 °C.

### Plasmid construction

To construct the overexpression vector, the full-length coding sequences (CDS) of ZIP2 was cloned into the Pro35S:pCAMBIA-1300GFP and Pro35S:pCAMBIA1307FLAG vector. The 1,407 bp ZIP2 promoter and 1,550 bp genomic sequence were fused to the pCAMBIA-1300 vector for the genetic complementation analysis. Mutagenesis of ZIP2^S190A^ and ZIP2^S190D^ was performed using the Tiangen Rapid Site-Directed Mutagenesis Kit (Tiangen, Beijing, China).

The ZIP2 CDS was cloned into the Pro35S:cYFP and Pro35S:pCAMBIA1300-cLUC vectors, respectively (Su et al. 2021). CIPK3/9/23/26-nYFP and CIPK3/9/23/26- nLUC were described previouslyand (Ju et al. 2022). The nYFP/cYFP/nLUC/cLUC labels all preceded the CDS.

To construct the recombinant protein vectors, ZIP2-Loop (451–609 bp) were amplified and cloned into the pGEX4T-1 vectors to obtain GST- ZIP2-Loop (Chen et al. 2021). CIPK3/9/23/26-GST were described previouslyand (Ju et al. 2022).

### Elemental analysis

The seeds of WT, *zip2*, *zip2*/ZIP2, *zip2*/ZIP2^S190A^ and *zip2*/ZIP2^S190D^ were germinated and grown on Hoagland medium and -Zn medium for 10 days, respectively. And then the roots and shoots were sampled. The samples were dried at 65 °C for 1 week. After that, the plant samples were digested in pure nitric acid solution at 120 °C, and 1 mL of H_2_O_2_ was added at the same time. Then the temperature was increased to 160 °C for 1 h, and then 2 mL of H_2_O_2_ was added to the digestion tube twice. Then keep it until the nitric acid is completely volatilized. Finally, the samples were diluted with ddH_2_O and analyzed by ICP-MS. Yeast cells with different vectors were cultured on SD-U and SD-U + 2 Mm EGTA liquid medium for 48 h, centrifuged at 700 g for 5 min, and washed three times with ddH_2_O. The samples were dried and digested according to the above method.

### Bimolecular fluorescence complementation assay

The bimolecular fluorescence complementation (BiFC) assay was based on a previously published method (Ju et al. 2022). CIPK3/9/23/26-nYFP and ZIP2-cYFP constructs were transiently expressed in *N.benthamiana* leaves. At 48 h after transfection, the YFP fluorescence signal in tobacco leaves was detected (IX83-FV3000; olympus).

### Split-luciferase complementation imaging assay

Luciferase complementary imaging (LCI) detection was performed according to the previous description (Su et al. 2021; Ju et al. 2024). Agrobacterium cells containing CIPK3/9/23/26-pCAMBIA1300-nLUC + ZIP2-pCAMBIA1300-cLUC (GV3101) were infiltrated into *N.benthamiana* leaves and expressed for 48 h. The signals were detected by a charge-coupled device (CCD; lumazone Pylon2048B; princeton, Trenton, NJ, USA).

### In vitro pull-down assay

In vitro pull-down experiments were performed as described above (Zhang et al. 2021). Because ZIP2 is a transmembrane protein containing 9 transmembrane regions, we have not successfully purified the full-length ZIP2 protein. Therefore, the largest Loop domain (151–203 aa) in the middle of ZIP2 was selected and named ZIP2-Loop. CIPK3/9/23/26 and ZIP2-Loop were amplified and cloned into pET30a or pGEX4T-1 vector to obtain CIPK3/9/23/26-His and GST-ZIP2-Loop. GST-ZIP2-Loop fusion protein was incubated with glutathione beads and then incubated with His-CIPK3/9/23/26. After elution, immunoblotting analysis was performed with anti-His antibody to detect His-CIPK3/9/23/26.

### Co-immunoprecipitation assay

Co-immunoprecipitation (Co-IP) assay was performed according to previously published methods (Ju et al. 2022). The total proteins of 10-day-old GUS-FLAG/ZIP2-GFP and CIPK26-FLAG/ZIP2-GFP transgenic seedlings were extracted with IP buffer (50 mM Tris HCl (pH 7.6), 150 mM NaCl, 10% glycerol, 5 mM MgCl_2_, 0.5% NP-40, 1 mM phenylmethylsulfonyl fluoride, 1 × protease inhibitor cocktail (Roche) and 1 mM DTT). Anti-GFP agarose beads (A2220; sigma-Aldrich), washed three times with PBS buffer, eluted with 5 × SDS-PAGE loading buffer, and then subjected to SDS-PAGE immunoblotting analysis. CIPK26 and ZIP2 were detected with anti-FLAG antibodies and anti-GFP antibodies (F1804; Sigma).

### In vitro and in vivo kinase assays

In vitro kinase assay was based on the published method (Zhang et al. 2021). ZIP2-Loop and mutant proteins were incubated with CIPK3/9/23/26-GST at 30 °C for 30 min in a kinase reaction buffer composed of 2.5 mM MgCl_2_, 20 mM Tris HCl (pH 7.2), 2.5 mM MnCl_2_, 1 mM DTT, 0.5 mM CaCl_2_, 50 mM ATP and 1 μCi (γ^32^-P) ATP, and then heated at 100 °C for 5 min in 5 × loading buffer. Subsequently, the proteins were separated by SDS-PAGE, and the signals were detected with a Typhoon 9410 imager (Cytiva Sweden AB, Uppsala, Sweden).

For the in vivo protein kinase assay, CIPK26-FLAG, ZIP2-FALG and ZIP2^S190A^-FLAG constructs were transformed into the *zip2* or *cipk3/9/23/26* mediated by *A. tumefaciens*. The transgenic materials were first grown on Hoagland’s solution for 7 days, then rinsed with ddH_2_O (to remove surface Zn^2+^ from the seedlings) and subsequently transferred to a -Zn medium for Zn-deficiency treatments lasting 0, 6, 12, 24, 72, and 120 h. The proteins were extracted and enriched with anti-FLAG agarose beads (Proteintech). The phosphorylation signals were analyzed by Western blot using a phosphoserine antibody (Immuno Way Biotechnology).

### Yeast functional analysis

ZIP2 and its variants were cloned into yeast expression vector pYES2 and transformed into yeast strain *Δzrt1/zrt2*. Transgenic yeasts were cultured in SD/-Ura medium for 5–7 days and cultured in SD/-Ura liquid medium to OD_600_ = 0.1. Under sterile conditions, four tenfold gradient dilutions were established, and 2.5 μL of each gradient dilution was dropped on SD/-Ura and SD/-Ura + 5 mM EGTA medium, respectively. The A_600_ values of the transgenic yeast were recorded every 3 h after 18 h of growth with SD/-Ura or SD/-Ura + 2 mM EGTA to prepare the growth curve.

### Western blot assay

The 7-day-old 35S:ZIP2-FLAG and 35S:ZIP2^S190A^-FLAG and transgenic plants were transferred to -Zn medium or -Zn medium supplemented with 10 μM MG132/CHX (Sigma-Aldrich, #474,790/#239,763-M) for 0, 6, 12, 24, 72 and 120 h, and the total protein was extracted. The protein loading buffer was added to the sample and heated at 95 °C for 10 min to denature the protein. The samples were subjected to sodium dodecyl sulfate–polyacrylamide gel electrophoresis (SDS-PAGE). The PVDF membrane was cut into a suitable size and transferred protein for 2 h at a constant current of 200 mA. The membrane was incubated with 5% skimmed dry milk for 2 h, and the appropriate primary antibody/secondary antibody was added for 2 h. Finally, images were obtained using a chemiluminescence imager (Cell Signaling Technology, USA).

### Subcellular Localization of ZIP2

Transgenic plants with ZIP2-GFP, the phosphodead and phosphomimetic ZIP2 variants were grown vertically under Hoagland and Zn-deficiency conditions for 5 d. Fluorescence was observed using a confocal microscope (IX83-FV3000; Olympus) with the following excitation and detection wavelengths, 488 and 500–540 nm for GFP and 561 and 650–710 nm for PI.

### Statistical analysis

The statistical significance of differences between mean values was determined using Student’s *t*-tests or ANOVA with Tukey’s multiple comparison test. Different asterisks against error bars of histograms are used to indicate means that are statistically different at *P* < 0.05. ‘n’ indicates the number of biological replicates.

## Supplementary Information

Supplementary Material 1.

## Data Availability

All relevant data can be found within the manuscript and its supporting materials.

## Abbreviations

BiFC: Bimolecular fluorescence complementation
LCI: Split luciferase complementation
ICP-MS: Inductively coupled plasma-mass spectrometry
OD: Optical density
CDS: Coding sequence
Co-IP: Co-Immunoprecipitation
COM: Complementary plants
DTT: Dithiothreitol
EDTA: Ethylene diamine tetraacetic acid
EGTA: Ethylenebis tetraacetic acid
GFP: Green fluorescent protein
GST: Glutathione S-transferase
NP-40: Nonidet P-40
PAGE: Polyacrylamide gel electrophoresis
PBS: Phosphate buffered saline
PI: Propidiumiodide
PMSF: Phenylmethyl sulfuryl fluoride
Pro: Promoter
qRT-PCR: Quantitative real-time PCR
YFP: Yellow fluorescent protein
YPD: Yeast extract peptone dextrose medium
OE: Overexpression
pH: Pondus hydrogenii
A: Alanine
D: Aspartate
S: Serine
