# Supplementary material for: Calcium signaling-mediated phosphorylation controls zinc allocation in Arabidopsis
Source: Stress Biol. 2025 Dec 31;5(1):78. doi: 10.1007/s44154-025-00276-z (PMC12756210; doi:10.1007/s44154-025-00276-z)
Supplement: Supplementary file 1 — Supplementary Material 1. [file 44154_2025_276_MOESM1_ESM.docx]

**
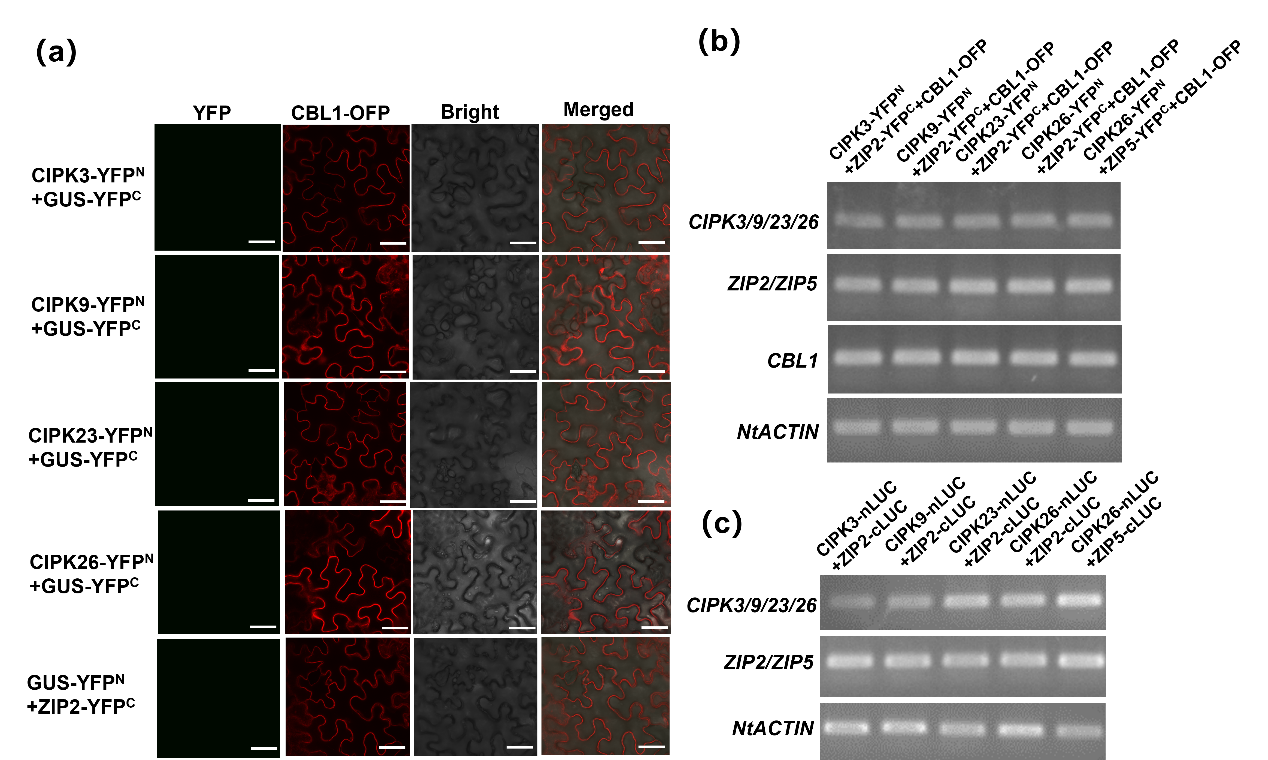
Supporting Information**

**Fig. S1. Semi-qRT-PCR analysis of BiFC and LCI.** (a) CIPK3/9/23/26-nYFP+ZIP2-cYFP, GUS-nYFP+ZIP2-cYFP vectors were constructed and co-expressed with CBL1-OFP in *Nicotiana benthamiana*. Bars, 40 μm. (b) Semi-qRT-PCR analysis of CIPK3/9/23/26, ZIP2/5, and CBL1 in the transiently infiltrated *N. benthamiana* leaves shown in (Figure 1a and Figure S1a). *NtActin* was used as a control. (c) Semi-qRT-PCR analysis of CIPK3/9/23/26, ZIP2/5 in the transiently infiltrated *N. benthamiana* leaves shown in (Figure 1b and Figure S1b). *NtACTIN* was used as a control.

**
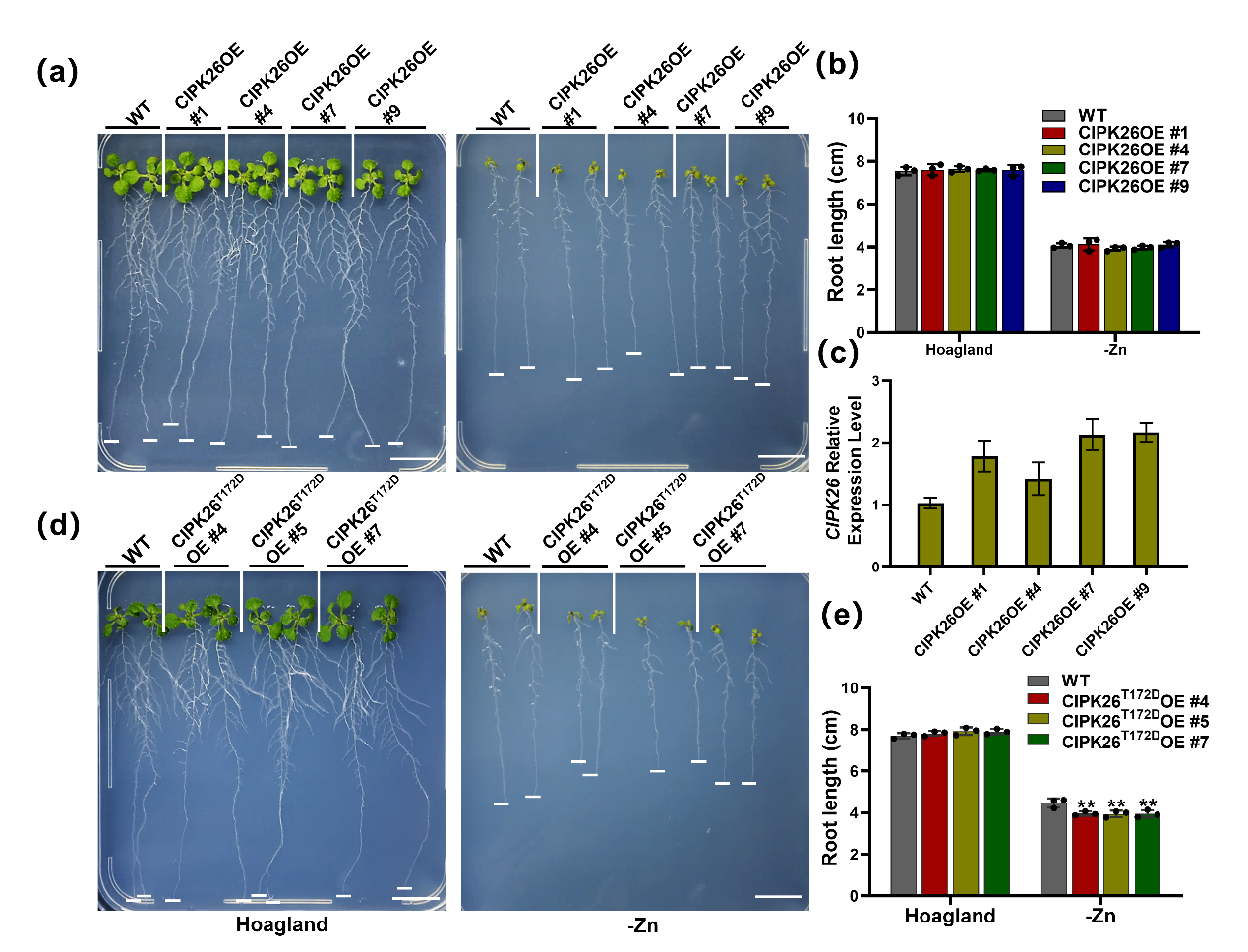
Fig. S2. Phenotypic analysis of CIPK26OE and CIPK26^T172D^OE in *Arabidopsis*.** (a) Zn deficiency phenotype of plants overexpressing CIPK26 plants. The plants were grown under Hoagland and -Zn conditions for 10 days. Scale bars, 1 cm. (b) Statistical analysis of root lengths of plants shown in (a). (c) The relative expression level of *CIPK26* in CIPK26OE plants. (d) Zn deficiency phenotype of plants overexpressing CIPK26^T172D^ plants. The plants were grown under Hoagland and -Zn conditions for 10 days. Scale bars, 1 cm. (e) Statistical analysis of root lengths of plants shown in (d). The data are presented as the mean ± SD (n = 16 seedlings for each genotype; Student’s *t*-test: *, *P* < 0.05 and **, *P* < 0.01).

**
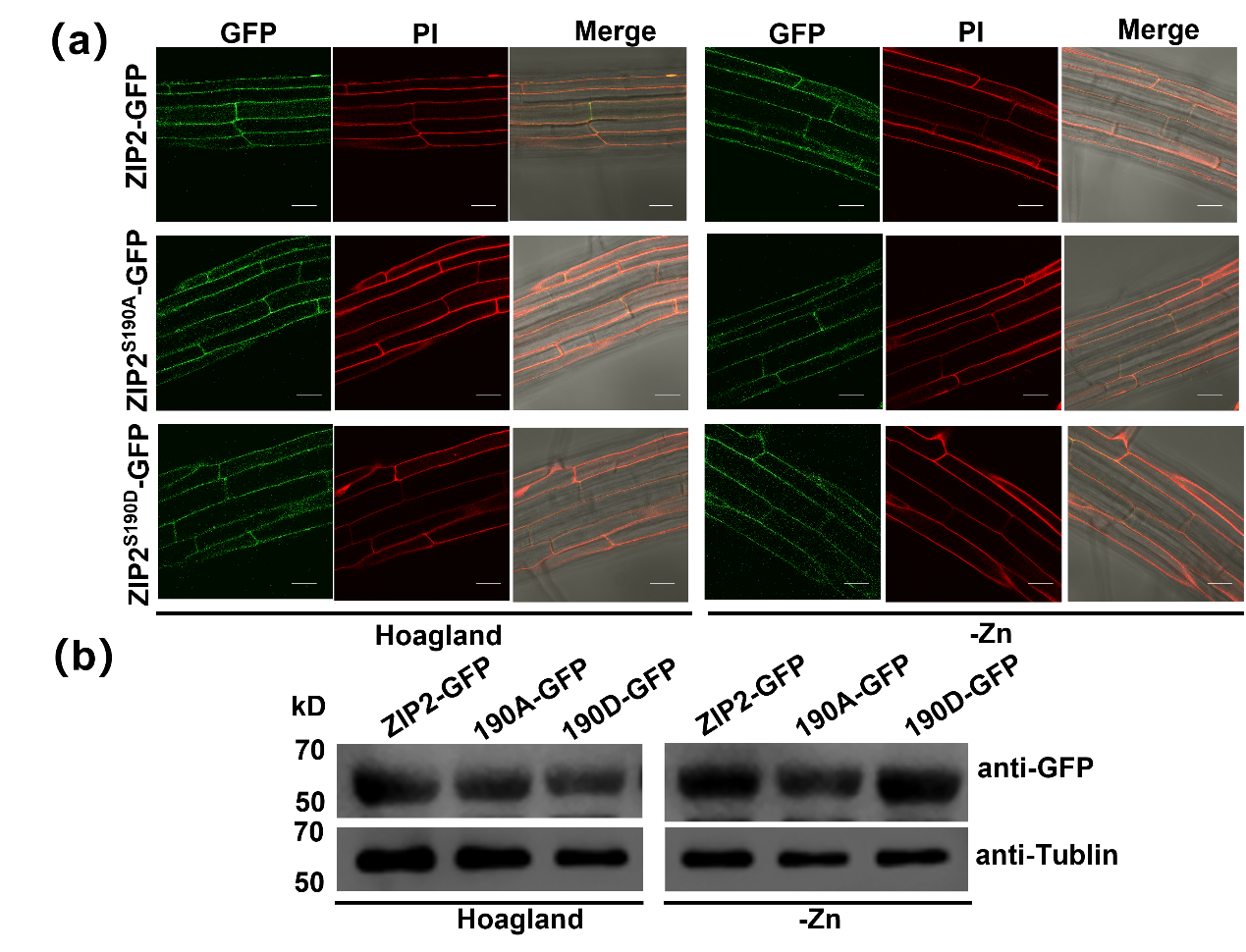
Fig. S3. CIPK3/9/23/26-mediated phosphorylation does not affect the subcellular localization of ZIP2 under Zn deficiency.** (a) The subcellular localization of the ZIP2, ZIP2^S190A^ and ZIP2^S190D^ were analyzed by confocal microscopy. The plants were grown for 5 days under Hoagland or -Zn conditions, and the root tip epidermal cells were observed under a confocal microscope. Scale bars, 10 µm. (b) Western blot analysis of ZIP2, ZIP2^S190A^ and ZIP2^S190D^ transgenic plants grown on Hoagland and -Zn medium. ZIP2, ZIP2^S190A^ and ZIP2^S190D^ protein levels were detected using anti-GFP antibody. Anti-Tubulin antibodies were used as a loading control.

**
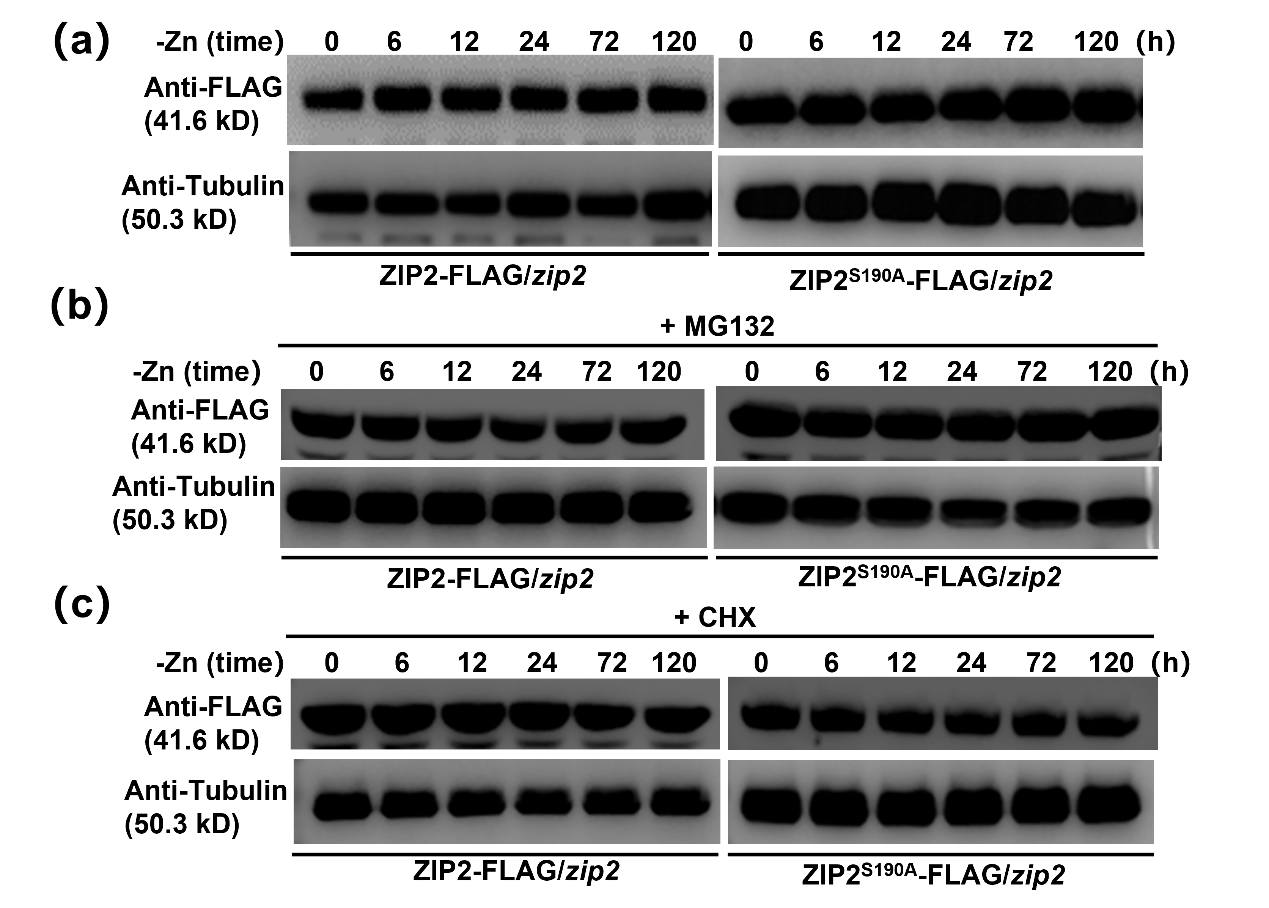
Fig. S4. CIPK3/9/23/26-mediated phosphorylation does not affect the protein accumulation of ZIP2 under Zn deficiency.** (a) Western blot analysis of ZIP2 and ZIP2^S190A^ in *zip2*. (b) Effect of MG132 treatment on ZIP2 and ZIP2^S190A^. (c) Effect of CHX treatment on ZIP2 and ZIP2^S190A^. ZIP2-FLAG/*zip2* and ZIP2^S190A^-FLAG/*zip2* transgenic plants grown under Hoagland conditions for 7 d were transferred to Zn-deficiency conditions or treated with MG132 and CHX for 0, 6, 12, 24, 72, 120 h. ZIP2 and ZIP2^S190A^ protein levels were detected using anti-FLAG antibodies. Tubulin were used as the reference protein.

**
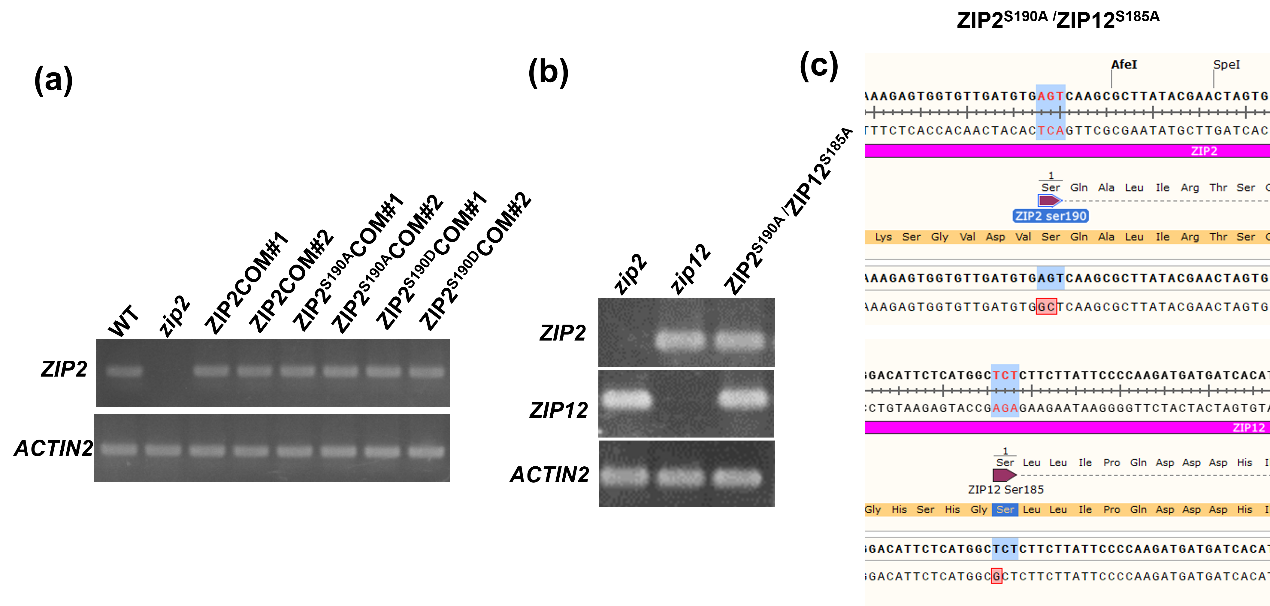
Fig. S5. Semi-qRT-PCR analysis of these transgenic plants.** (a) Semi-qRT-PCR analysis of the expression level of *ZIP2* in ProZIP2:ZIP2/*zip2*, ProZIP2:ZIP2^S190A^/*zip2* and ProZIP2:ZIP2^S190D^/*zip2* transgenic plants. (b) Semi-qRT-PCR analysis of the expression level of *ZIP2* and *ZIP12* in *zip2*, *zip12* and ZIP2^S190A^/ZIP12^S185A^ plants. RNA extracted from the roots of 10-day-old seedlings grown on 1/2 MS. *ACTIN2* is the reference gene. (c) Genotypic analysis of the transgenic plants ZIP2^S190A^/ZIP12^S185A^ confirmed that the codon for Ser190 in ZIP2 was successfully changed from AGT to GCT (encoding Ala), and similarly, the Ser185 codon in ZIP12 was mutated from TCT to GCT (Ala).


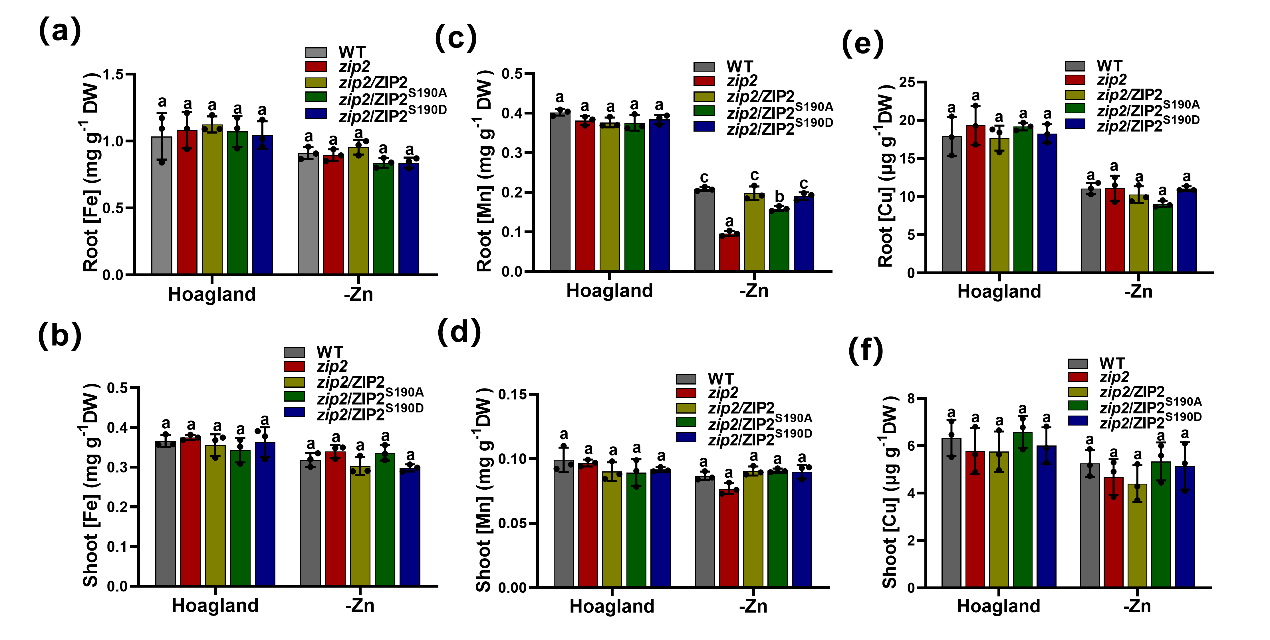
**Fig. S6. Fe, Mn and Cu concentration in WT and *zip2* and ProZIP2:ZIP2/*zip2*, ProZIP2:ZIP2^S190A^/*zip2* and ProZIP2:ZIP2^S190D^/*zip2* transgenic plants.** (a and b) Fe concentration in roots and shoots of these plants. (c and d) Mn concentration in roots and shoots of these plants. (e and f) Cu concentrationin roots and shoots of these plants. Bars represent the mean ± SD (n=3 independent biological replicates). Statistical differences were calculated by one-way ANOVA. Different letters indicate means that were statistically different by Tukey’s multiple testing method (*P* < 0.05) for genotypes within a given growth condition (Hoagland or -Zn).

**Supplemental Table S1. Primers used in this study.**

| **For genotyping** | **5’ —3’ sequence** |
| --- | --- |
| *cbl1*-SALK_110426-LP | GGGCTACGATACATTGAATCG |
| *cbl1*-SALK_110426-RP | TTGATCGTCTGGTTTCGAATC |
| *cbl4*-GK-015F02-LP | TTGGAATTCTCTATGGGCTGCTCTGTATC |
| *cbl4*-GK-015F02-RP | TTTTTGTGACTTAGGAAGATACGTTTTGC |
| *cbl5*-GK-278H02-LP | TTTGGATCCTGAATGGGATGTGTTTGCAGC |
| *cbl5*-GK-278H02-RP | TTTCCCGGGCTTCAAGAAAGGGATAGTCATGTT |
| *cbl8*-SALK_083553-LP | tttggatcccatgttggcattcgtgaaatg |
| *cbl8*-SALK_083553-RP | tttgaattcctagtcttcaacttcagagtcgagtac |
| *cbl9*-SALK_142774-LP | CCCCTTGCTAGTTGGGTTATC |
| *cbl9*-SALK_142774-RP | AAGAAGAAGCTTGTTCCGGAG |
| *cipk3*-SALK_137779-LP | ATCATCCACACAAAGTAGCCG |
| *cipk3*-SALK_137779-RP | TTTTTGGCAAAAATCAACCAC |
| *cipk9*-SALK_014699-LP | TTCAAACCGAGTTTGAGGATG |
| *cipk9*-SALK_014699-RP | TCCAAATTCTCCATCTGGTTG |
| *cipk23*-SALK_036154-LP | TTGTGATCCTCTTGCATAGGG |
| *cipk23*-SALK_036154-RP | AATCATCCCGGACAAAGTACC |
| *cipk26*-SALK_000085-LP | ATACTTTCCAACACGACGCTG |
| *cipk26*-SALK_000085-RP | GTGAAGAAGCAACGGTTTTTC |
| *zip2*-SALK_045578-LP | TGCCTATTTCGTAGCCATAGG |
| *zip2*-SALK_045578-RP | ACATCTGCCAGCATTGTAAGG |
| LBb1.3 (for SALK lines) | GCTTCCTATTATATCTTCCCAAATTACCAATACA |
| LB-GABI (for GK lines) | GGGCTACACTGCATTGGTAGCTC |
| **For cloning 5’ —3’ sequence** | |
| ZIP2-COM-F | gggcccggcgcgccgaattctattacctcttcttcaatttaaaaggt |
| ZIP2-COM-R | ggtggactcctcttaaagcttgtcgaatctaattgttctagcacata |
| ZIP2-FLAG-F | CAGCAATTAAGCTTGTCGACATGGCTTTGTCTTCCAAAACCCT |
| ZIP2-FLAG-R | CCGCCACCGCGGTGGAGCTCATCCCAAATCATTACAACAGAGAGC |
| ZIP2-GFP-F | AGAACACGGGGGACTCTAGAATGGCTTTGTCTTCCAAAACCCT |
| ZIP2-GFP-R | ACCATGGTACCCGGGGATCCATCCCAAATCATTACAACAGAGAGC |
| ZIP2-PYES2-F | CCGCCAGTGTGCTGGAATTCATGGCTTTGTCTTCCAAAACCCT |
| ZIP2-PYES2-R | ACATGATGCGGCCCTCTAGATCAATCCCAAATCATTACAACAGAGAGC |
| ZIP2-CE-F | aggcctggcgcgccactagtATGGCTTTGTCTTCCAAAACCCTAAAG |
| ZIP2-CE-R | tacatcccgggagcggtaccATCCCAAATCATTACAACAGAGAGC |
| ZIP2-cLUC-F | acgcgtcccggggcggtaccATGGCTTTGTCTTCCAAAACCCTAAA |
| ZIP2-cLUC-R | tccatttgttggatccTCAATCCCAAATCATTACAACAGAGAGCAAAG |
| CIPK3-GST-F | tggatccccggaattcATGAATCGGC CAAAGGTTCA GCGTC |
| CIPK3-GST-R | cggccgctcgagtcgacTTATTTGCTT AGACCAGAGC TCTCG |
| CIPK9-N-GST-F | tggatccccggaattcATGGAAATGAGTGGAAGCAGAAGGAAG |
| CIPK9-N-GST-R | cggccgctcgagtcgacTTGCTTTTGTTCTTCAGCGGC |
| CIPK23-GST-F | CCGCGTGGATCCCCGGAATTCATGGCTTCTCGAACAACGCCT |
| CIPK23-GST-R | GATGCGGCCGCTCGAGTCGACTTATGTCGACTGTTTTGCAAT |
| CIPK26-GST-F | TGGATCCCCGGAATTCATGAATCGGCCAAAGGTTCAGCGTC |
| CIPK26-GST-F | CGGCCGCTCGAGTCGACTTATTTGCTTAGACCAGAGCTCTCG |
